# ﻿Three new species of the genus *Zaitzevia* Champion, 1923 (Coleoptera, Elmidae) from China

**DOI:** 10.3897/zookeys.1174.101046

**Published:** 2023-08-10

**Authors:** Ri-Xin Jiang, Xiang-Sheng Chen

**Affiliations:** 1 Institute of Entomology, Guizhou University, Guiyang 550025, Guizhou, China; 2 The Provincial Special Key Laboratory for Development and Utilization of Insect Resources of Guizhou, Guizhou University, Guiyang, 550025, Guizhou, China; 3 The Provincial Key Laboratory for Agricultural Pest Management of Mountainous Region, Guiyang 550025, Guizhou, China

**Keywords:** China, Elmidae, Macronychini, new species, riffle beetle

## Abstract

Three new riffle beetles of the genus *Zaitzevia* Champion, 1923 are described from China, namely *Zaitzeviasichuanensis***sp. nov.** and *Zaitzeviafengtongzhaiensis***sp. nov.** from Sichuan Province, and *Zaitzeviayingzuijieensis***sp. nov.** from Hunan Province. Habitus and diagnostic features of the new species are illustrated. A checklist of all known Chinese *Zaitzevia* species is given, and a key and distributional map of *Zaitzevia* species from the Chinese mainland are provided.

## ﻿Introduction

The Macronychini genus *Zaitzevia* Champion, 1923 includes 24 valid species that are distributed from East, Southeast, and Central Asia to North America (Jäch et al. 2016; Jiang and Wang 2021; Bian and Zhang 2022). Recently, six species were described from China (Jiang and Wang 2020, 2021; Bian and Zhang 2022), and the Japanese fauna of *Zaitzevia* was also reviewed (Iwata et al. 2022), with its diversity still unclear and numerous species are still undescribed (Jäch and Boukal 1995). Before this study, 10 species of the genus were recorded from China (Jäch and Boukal 1995; Jäch et al. 2016; Jiang and Wang 2020, 2021; Bian and Zhang 2022).

Here, we add three new species of *Zaitzevia* to the Chinese fauna: *Zaitzeviasichuanensis* sp. nov. and *Zaitzeviafengtongzhaiensis* sp. nov. both from Sichuan Province, and *Zaitzeviayingzuijieensis* sp. nov. from Hunan Province. Diagnoses, descriptions, and illustrations of the new species are provided. A list of all known Chinese *Zaitzevia* species (Table 1), key, and a distributional map (Fig. 8) of mainland Chinese *Zaitzevia* species are also provided.

**Table 1.** List of known Chinese *Zaitzevia* species.

Species	Distribution
*Zaitzeviaformosana* Nomura, 1963	Taiwan
*Zaitzeviababai* Nomura, 1963	Taiwan
*Zaitzeviaparallela* Nomura, 1963	Taiwan
*Zaitzeviatsushimana* Nomura, 1963	Jilin; Japan; Korea; Russia
*Zaitzeviachenzhitengi* Jiang & Wang, 2020	Sichuan, Yunnan, Shannxi
*Zaitzeviaxiongzichuni* Jiang & Wang, 2020	Yunnan
*Zaitzeviatangliangi* Jiang & Wang, 2021	Hubei
*Zaitzeviamuchenae* Bian & Zhang, 2022	Yunnan
*Zaitzeviareniformis* Bian & Zhang, 2022	Yunnan
*Zaitzeviagaoligongensis* Bian & Zhang, 2022	Yunnan
*Zaitzeviasichuanensis* sp. nov.	Sichuan
*Zaitzeviafengtongzhaiensis* sp. nov.	Sichuan
*Zaitzeviayingzuijieensis* sp. nov.	Hunan

## ﻿Materials and methods

The material examinedduring this work is deposited in the Institute of Entomology, Guizhou University, Guiyang, China (**GUGC**).

Collecting data of the specimens are quoted verbatim. The Chinese translation of each locality below provincial level is included in parentheses at the first appearance in the text. Each type specimen bears the following label: ‘HOLOTYPE (red) (or PARATYPE (yellow)), ♂, *Zaitzevia* + specific name sp. nov., Jiang & Chen, 2023’.

Habitus images were taken using a Canon 5D Mark IV digital camera with an MP-E 65 mm f/2.8 1–5× macro lens. A Godox MF12 flash was used as the light source. Images of the morphological details were taken using a Canon 5D Mark IV digital camera in conjunction with a Mitutoyo Plan NIR 10 lens and a Godox MF12 flash was used as the light source or a Nikon SMZ25 stereoscopic microscope with a Nikon DS-Ri2 camera. Zerene Stacker (v. 1.04) was used for image stacking. All images were improved and grouped into plates in Adobe Photoshop CS5 Extended.

Morphological terminology and the format for the descriptions follow those of Jiang and Wang (2021). The following abbreviations are used in the text: **HL**—length of head from the anterior epistomal margin to the occipital constriction; **HW**—width of head across compound eyes; **PL**—length of pronotum along the midline; **PW**—maximum width of pronotum; **EL**—length of elytra along the suture; **EW**—maximum width of elytra; **CL**—the sum of PL + EL.

## ﻿Taxonomy

### ﻿ Zaitzevia sichuanensis

Taxon classificationAnimaliaColeopteraElmidae

Jiang & Chen sp. nov.

7786F923-FE6B-5994-9C3E-4849230656AD

https://zoobank.org/7E78CB26-B54D-447B-B110-CCCEACA0635A

**Figure 1.**
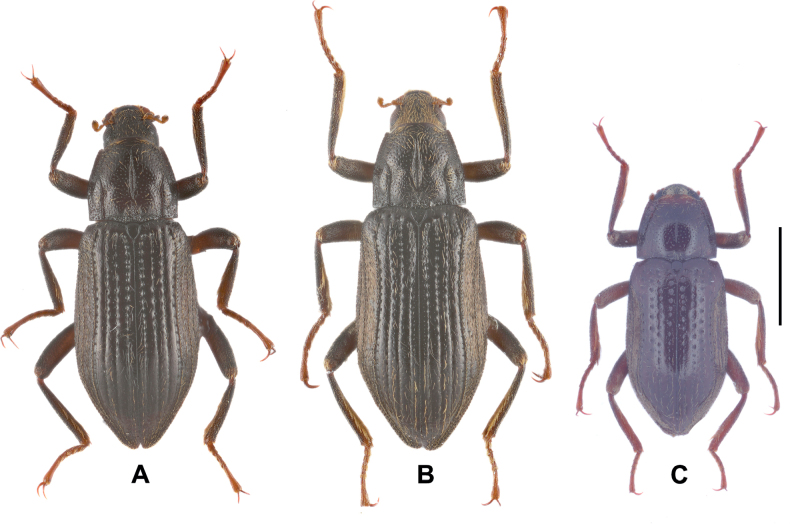
Dorsal habitus of *Zaitzevia* species, males **A***Zaitzeviasichuanensis* sp. nov. **B***Zaitzeviafengtongzhaiensis* sp. nov. **C***Zaitzeviayingzuijieensis* sp. nov. Scale bar: 1 mm.

**Figure 2.**
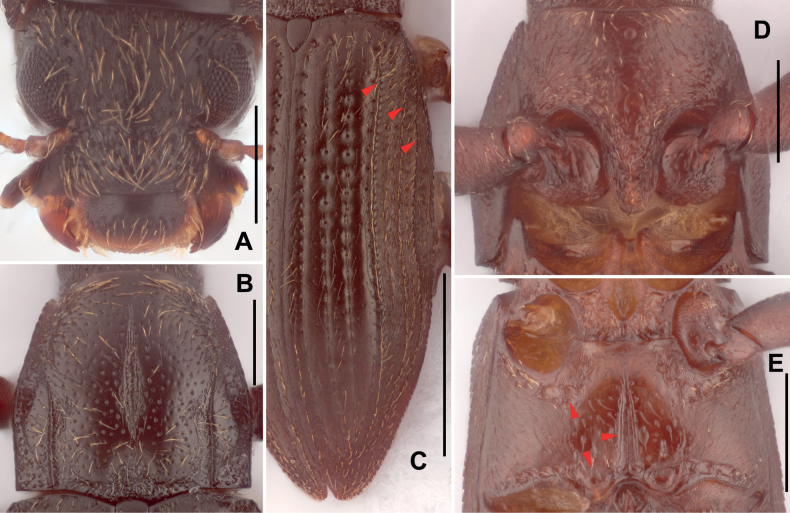
Diagnostic features of *Zaitzeviasichuanensis* sp. nov. **A** head, dorsal view **B** pronotum, dorsal view **C** elytra **D** prosternal process **E** metaventrite. Scale bars: 1 mm (**C**); 0.25 mm (**A, B, D, E**). Note: red arrow of **C** granulate carinae of elytra; red arrow of **E** impressions and median sulcus of metaventrite.

**Figure 3.**
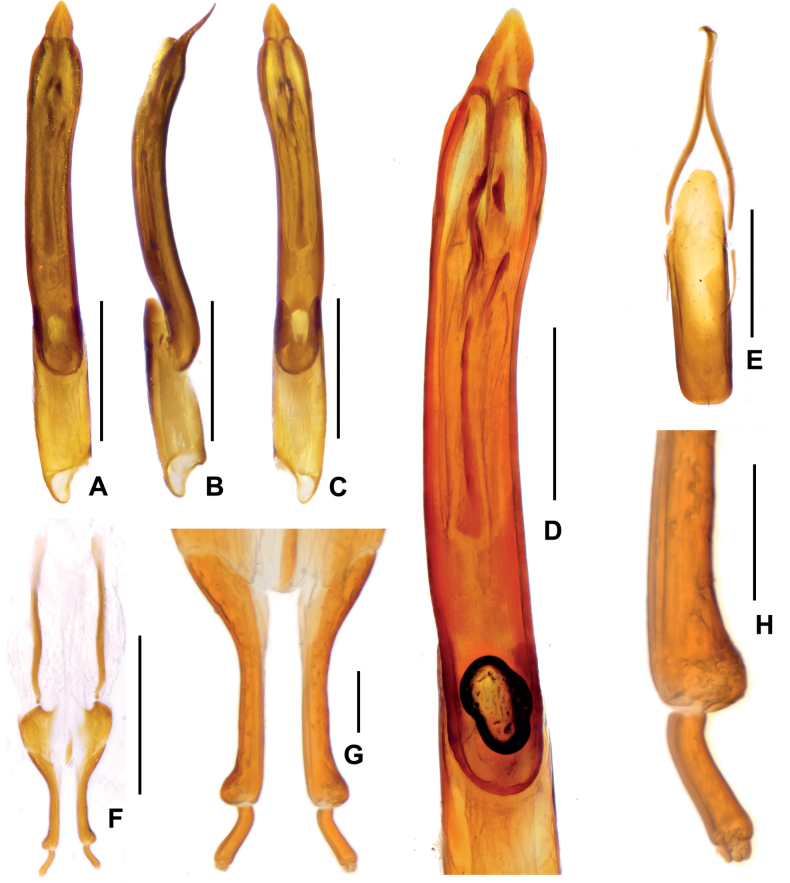
Diagnostic features of *Zaitzeviasichuanensis* sp. nov. **A** aedeagus, dorsal view **B** ditto, lateral view **C** ditto, ventral view **D** ditto, meidan lobe, dorsal view **E** dternite IX **F** ovipositor **G, H** apical part of ovipositor. Scale bars: 0.5 mm (**A–C, E, F**); 0.2 mm (**D, G**); 0.1 mm (**H**).

Figs 1A , 2 , 3 , 9A–D 川寥溪泥甲

#### Type material

**(2** ♂♂, **2** ♀♀) **. *Holotype***: China: ♂, labeled ‘China: Sichuan, Ya’an City (雅安市), Muping Town (穆坪镇), Lengmugou Geopark (冷木沟地质公园), an unnamed stream, 30°22′25′′N, 102°48′52′′E; H: 935m, 25.07.2022, R.-X. Jiang & F.-E. Li leg.’ (GUGC). ***Paratype***: 1 ♂, 2 ♀♀; 1 ♀, with the same label data as the holotype; 1 ♂, 1 ♀, labeled ‘Sichuan, Chengdu City (成都市), Dayi County (大邑县), Xiling Town (西岭镇), Xilingxueshan (西岭雪山), Jiaoziping (椒子坪), an unnamed stream, 30°40′12′′N, 103°15′07′E; H: 1350m, 21.07.2022, R.-X. Jiang & F.-E. Li leg.’ (GUGC).

#### Description.

**Male. *Body elongately elliptical*** (Fig. 1A), black, with tarsi, tarsal claws and antennae reddish brown, tibiae brown. Dorsal surface punctuate and weakly shiny, covered with sparse short setae. Plastron setae is confined to following areas: head (both dorsal and ventral surface), prosternum, outer part of elytra (include epipleura), outer parts of mesoventrite, metaventrite and abdomen (except median part) and surface of femora.

***Head*** (Fig. 2A) wider than long, dorsal surface covered with dense short setae and large, sparse punctures, each puncture bearing a long seta, the interspaces between the punctures about 1.5–2 times of the diameters of punctures. Clypeus evenly punctate with large punctures and covered with long, sparse setae. Labrum transverse, shorter and slightly narrower than clypeus, covered with large punctures and long bristles at apical portion, anterior margin almost straight and anterolateral angles rounded. Antenna short, with eight antennomeres, antennomere I slightly longer than wide, with several short setae; antennomere II about as long as antennomere I, distinctly expanded, covered with several long setae, apical margin circled with short setae; antennomere III longer than wide; antennomeres IV–VII strongly transverse; antennomere VIII elliptical, elongate and strongly expanded, apex covered with long, dense setae.

Pronotum (Fig. 2B) slightly wider than long, widest near basal 2/5. Anterior margin arcuate with angles moderately produced and acute. Lateral margins finely curved. Basal margin trisinuate, emarginate before scutellum, posterior angles obtuse. Surface shiny, finely covered with large punctures, each puncture bearing a long seta; surface near anterior angles microreticulate. Distinct longitudinal impression in the middle of the pronotum, basal 1/3 much wider than other parts; sublateral carinae from base to middle of pronotum, apical 1/2 curved, lateral parts of sublateral carinae distinctly convex. Prosternal process (Fig. 2D) with rounded apex, disc without plastron setae, surface distinctly wrinkled.

***Elytra*** (Fig. 2C) about twice as long as wide, subparallel in basal 3/5, surface weakly wrinkled and covered with long, sparse setae. Each elytron with granulate carinae on strial intervals 5, 7, and 8; other intervals flat. Area from intervals to lateral margin covered with short, dense setae. Hind wings well developed.

Metaventrite (Fig. 2E): surface of disc smooth, covered with large, sparse punctures, each bearing a long seta, sides partly covered with plastron setae. Median sulcus distinct, extending in posterior ca. 3/4, narrower and shallower from base to apex, base of median sulcus with a pair of small round impressions. Areas along coxal cavities with a series of shallow and anomalous impressions.

***Disc of ventrites I–IV*** and anteriorly middle of ventrite V shiny, covered with small, sparse punctures, without plastron setae; other areas of ventrites covered with plastron setae. Apical area of ventrite V granulated, apical margin distinctly emarginate at middle.

***Legs*** simple, femora swollen, surface covered with plastron setae; inner side of distal halves of tibiae with cleaning fringes; tarsi slightly shorter than tibiae; tarsal claws simple and strong.

***Aedeagus*** (Fig. 3A–D), slender and elongate, apex of median lobe asymmetrically arrowhead-like and weakly curved at middle, with a pair of short sclerotizations located at apical 1/5 and a pair of longer sclerotizations near the short sclerotizations, a much longer sclerotization located at middle of median lobe. Sternite IX (Fig. 3E) with a tuft of short setae at middle of apical margin, paraproct with base slightly expanded and tortuous.

***Measurements***: CL: 3.29–3.35 mm; HL: 0.41–0.43 mm, HW: 0.56–0.59 mm; PL: 0.85–0.86 mm, PW: 0.91–0.97 mm; EL: 2.44–2.50 mm, EW: 1.24–1.32 mm.

**Female**: externally similar to the male, apex of sternite VIII rounded. Ovipositor as in Fig. 3F–H, stylus weakly curved at base, apex with three short finger-like sensilla; apex of coxite roundly broadened at outer margin, without sensilla; valvifer longer than coxite, fibula weakly sinuate with basal 1/5 expanded. Measurements: CL: 3.20–3.26 mm; HL: 0.42–0.47 mm, HW: 0.54–0.56 mm; PL: 0.82–0.83 mm, PW: 0.92–0.93 mm; EL: 2.38–2.43 mm, EW:1.22–1.24 mm.

#### Distribution.

China: Central Sichuan Province.

#### Biology.

All adults were collected from bottom crack of stone in small ravine stream (Fig. 9A–D).

#### Etymology.

The specific epithet refers to the type locality, Sichuan Province; the name is treated as an adjective.

#### Comparative diagnosis.

*Zaitzeviasichuanensis* sp. nov. is more or less similar to *Zaitzeviachenzhitengi* Jiang & Wang, 2020 from Sichuan Province and *Zaitzeviamuchenae* Bian & Zhang, 2022 from Yunnan Province. All three species share similar habitus, e. g. the relatively large and elongate oval body shape (both species > 3mm) and the wrinkled elytra. However, the new species can be well distinguished from *Z.chenzhitengi* by the following characters: 1) elytra weakly wrinkled (cf. distinctly wrinkled in *Z.chenzhitengi*); 2) different form of elytra (wider in the new species of male, 1.24–1.32 mm in the new species, 1.06 mm in *Z.chenzhitengi*); 3) different form of aedeagus (both two species with apex of median lobe arrowhead-like, but the median lobe shorter in *Z.chenzhitengi*, but much slender and with median lobe widely arrowhead in the new species); 4) different modification of areas between the lateral margins and the sublateral carinae (distinctly convex in the new species, and not convex in *Z.chenzhitengi*). The new species can be distinguished from *Z.muchenae* by the much longer and wider longitudinal impression of pronotum and the obviously different form of aedeagus.

### ﻿ Zaitzevia fengtongzhaiensis

Taxon classificationAnimaliaColeopteraElmidae

Jiang & Chen sp. nov.

9D122626-BC16-5C9A-BB17-AB875196363E

https://zoobank.org/A32F5E97-91DB-41D0-9A7E-F243CC0B86AD

**Figure 4.**
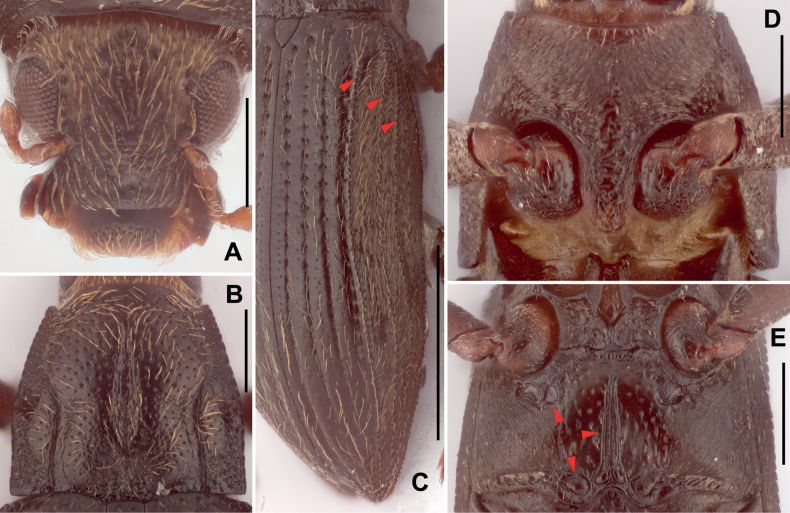
Diagnostic features of *Zaitzeviafengtongzhaiensis* sp. nov. **A** head, dorsal view **B** pronotum, dorsal view **C** elytra **D** prosternal process **E** metaventrite. Scale bars: 1 mm (**C**); 0.25 mm (**A, B, D, E**). Note: red arrow of **C** granulate carinae of elytra; red arrow of **E** impressions and median sulcus of metaventrite.

**Figure 5.**
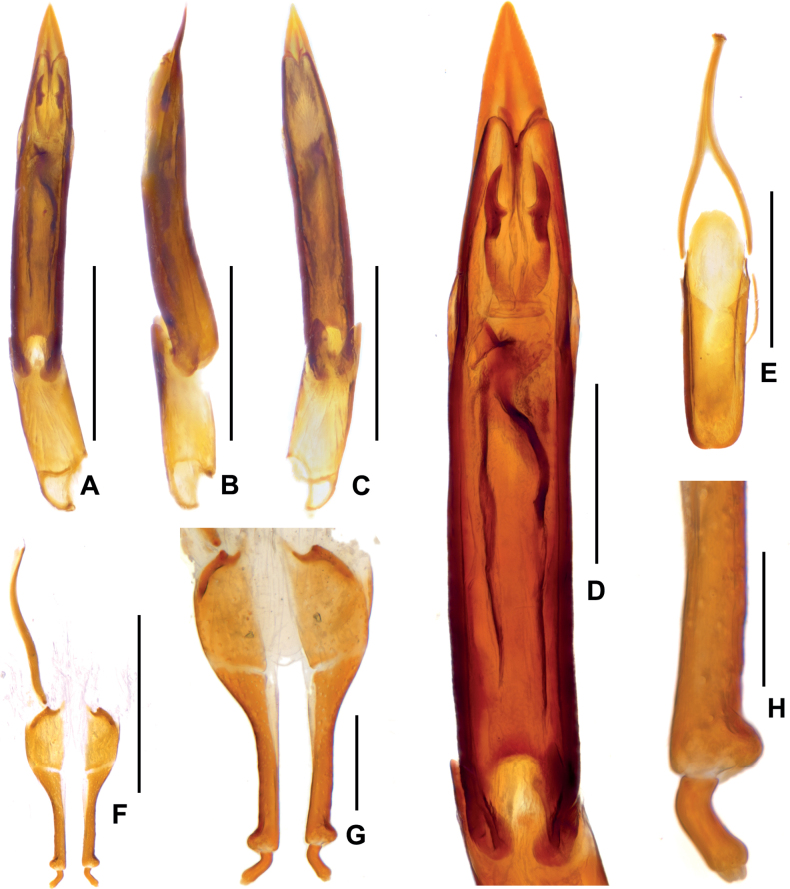
Diagnostic features of *Zaitzeviafengtongzhaiensis* sp. nov. **A** aedeagus, dorsal view **B** ditto, lateral view **C** ditto, ventral view **D** ditto, meidan lobe, dorsal view **E** sternite IX **F** ovipositor **G, H** apical part of ovipositor. Scale bars: 0.5 mm (**A–C, E, F**); 0.2 mm (**D, G**); 0.1 mm (**H**).

Figs 1B , 4 , 5 , 9E–G 蜂桶寨寥溪泥甲

#### Type material

**(1** ♂, **1** ♀) **. *Holotype***: China: ♂, labeled ‘China: Sichuan, Ya’an City (雅安市), Baoxing County (宝兴县), Fengtongzhai Township (蜂桶寨乡), Fengtongzhai N. R. (蜂桶寨国家级自然保护区), Dashuigou (大水沟), an unnamed stream, 30°35'18′′N, 102°52′24′′E; H: 1505m, 23.07.2022, R.-X. Jiang & F.-E. L. leg.’ (GUGC). ***Paratype***: 1 ♀, with the same label data as the holotype (GUGC).

#### Description.

**Male. *Body elongately elliptical*** (Fig. 1B), black with tarsi, tarsal claws and antennae reddish brown, femora and tibiae dark brown. Dorsal surface punctuated and weakly shiny, covered with sparse setae. Plastron setae are confined to following areas: head (both dorsal and ventral surface, except middle part of frons and clypeus), prosternum, outer part of elytra (include epipleura), outer parts of mesoventrite, metaventrite and abdomen (except median part) and surface of femora.

***Head*** (Fig. 4A), wider than long, dorsal surface (except middle part of frons) covered with plastron setae and large, sparse punctures, each puncture bearing a long seta, the interspaces between the punctures about twice the diameters of punctures. Clypeus evenly punctate with large punctures and covered with long, sparse setae, without plastron setae. Labrum transverse, shorter and slightly narrower than clypeus, covered with big punctures and long bristles at apical half, anterior margin almost straight and anterolateral angles rounded. Antenna short, with eight antennomeres, antennomere I slightly longer than wide, with several short setae; antennomere II slightly longer than antennomere I, strongly expanded, covered with several long setae, apical margin circled with short setae; antennomere III longer than wide; antennomeres IV–VII strongly transverse; antennomere VIII elliptical, elongate and strongly expanded, apex covered with dense long setae.

***Pronotum*** (Fig. 4B) wider than long, widest near basal 1/3. Anterior margin arcuate with angles moderately produced and acute. Lateral margins finely curved. Basal margin trisinuate, emarginate before scutellum, posterior angles near orthogonal. Surface shiny, finely covered with large punctures, each puncture bearing a long seta, punctures at basal 1/3 smaller and much denser than other parts; surface near apical angles microreticulate. Longitudinal impression distinct, about 2/3 length of pronotum, widest at middle; sublateral carinae from base to middle of pronotum, apical 1/2 slightly curved. Prosternal process (Fig. 4D) with rounded apex, disc distinctly wrinkled, sides microreticulated.

***Elytra*** (Fig. 4C) about twice as long as wide, subparallel in basal 1/3, surface weakly wrinkled and covered with rows of long, sparse setae. Each elytron with granulate carinae on strial intervals 5, 7, and 8; other intervals flat. Area from intervals to lateral margin covered with dense short setae. Hind wings well developed.

***Metaventrite*** (Fig. 4E), disc shiny, covered with large, sparse punctures, each bearing a long seta, without plastron setae, sides covered with plastron setae. Median sulcus long and distinct, extending from posterior margin to ca. 4/5 of metasternum, widest at base and get narrowed anteriorly, base of median sulcus with a pair of small round impressions. Areas along coxal cavities with a series of shallow and anomalous impressions.

***Disc of ventrites I–IV*** and anteriorly middle of ventrite V shiny, covered with sparse small punctures, without plastron setae; other areas of ventrites covered with plastron setae. Apical area of ventrite V granulated, apical margin distinctly emarginate at middle.

***Legs*** simple, femora swollen, surface covered with plastron setae; inner half of tibiae with cleaning fringes; tarsi slightly shorter than tibiae; tarsal claws simple.

***Aedeagus*** (Fig. 5A–D), slender and elongate, median lobe nearly symmetrical, apex of median lobe acute, a pair of symmetrical and crescent-shaped sclerotizations located at apical 1/5 of median lobe, a pair of elongate and tortuous sclerotizations located at basal 1/2 of median lobe. Sternite IX (Fig. 5E) with apical margin weakly emarginate, without seta, paraproct with basal slightly expanded and tortuous.

***Measurements***: CL: 3.51 mm; HL: 0.47 mm, HW: 0.57 mm; PL: 0.87 mm, PW: 0.96 mm; EL: 2.64 mm, EW: 1.32 mm.

**Female**: externally similar to the male, apex of sternite V rounded. Ovipositor as in Fig. 5F–H, stylus distinctly curved at base, apex with three short, finger-like sensilla; apex of coxite strongly roundly broadened at outer margin, without sensilla; valvifer about as long as coxite, fibula distinctly curved at basal 1/2, base weakly expanded. Measurements: CL: 3.46 mm; HL: 0.45 mm, HW: 0.58 mm; PL: 0.84 mm, PW: 0.90 mm; EL: 2.62 mm, EW:1.33 mm.

#### Distribution.

China: Central Sichuan Province.

#### Biology.

All adults were collected from a crack in the bottom of a stone in a small ravine stream (Fig. 9E–G).

#### Etymology.

The specific epithet refers to the type locality, Fengtongzhai Nature Reserve, Sichuan Province; the name is treated as an adjective.

#### Comparative diagnosis.

*Zaitzeviafengtongzhaiensis* sp. nov. is similar to *Zaitzeviachenzhitengi* Jiang & Wang, 2020, *Zaitzeviasichuanensis* sp. nov. and *Zaitzeviamuchenae* Bian & Zhang, 2022, and all four of these species share a similar habitus. The new species can be well distinguished from *Z.chenzhitengi* by the following characters: 1) different body size (> 3.30mm in the new species, 3.03–3.20 mm in *Z.chenzhitengi*); 2) elytra weakly wrinkled (cf. distinctly wrinkled in *Z.chenzhitengi*); 3) obviously different form of aedeagus (apex of median lobe arrowhead-like in *Z.chenzhitengi*, but simply acute in the new species). Compared with *Z.sichuanensis* sp. nov., areas between lateral margins and sublateral carinae not convex in the new species, and distinctly convex in *Z.sichuanensis* sp. nov., these two species also can be distinguished by the obviously different from of aedeagus (see Fig. 4A–F). *Zaitzeviafengtongzhaiensis* sp. nov. can be distinguished from *Z.muchenae* by the following characters: 1) aedeagus of the new species is similar to *Z.xiongzichuni* Jiang & Wang, 2020, both species with a pair of crescent-shaped sclerotizations near apex of median lobe. However surface of *Z.xiongzichuni* shiny and not wrinkled. Median lobe of *Z.xiongzichuni* much slender, and weakly narrowed at middle, apical margin of sternite IX with a tuft of short setae, while median lobe of *Z.fengtongzhaiensis* sp. nov. is shorter and not narrowed at middle, and the apical margin of sternite IX is without setae.

### ﻿ Zaitzevia yingzuijieensis

Taxon classificationAnimaliaColeopteraElmidae

Jiang & Chen sp. nov.

DCFEC690-3CCC-5814-97AB-E0F3766544E7

https://zoobank.org/57149AEE-921A-4AF3-9DAC-8BDC9BF37239

**Figure 6.**
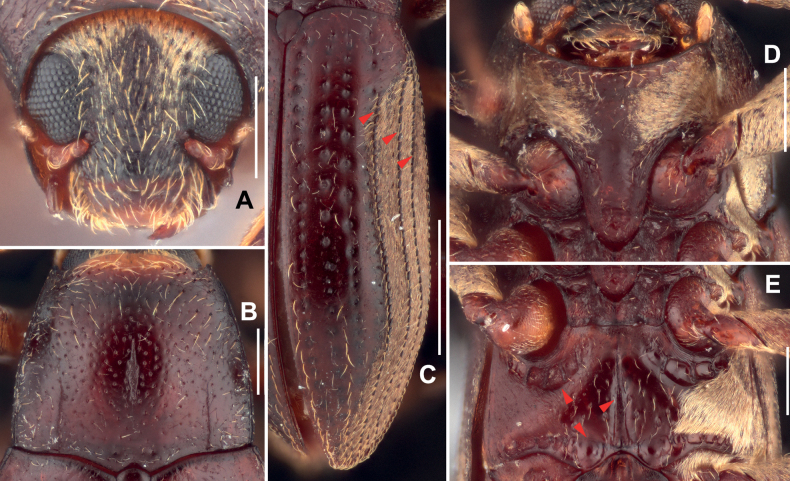
Diagnostic features of *Zaitzeviayingzuijieensis* sp. nov. **A** head, dorsal view **B** pronotum, dorsal view **C** elytra **D** prosternal process **E** metaventrite. Scale bars: 1 mm (**C**); 0.25 mm (**A, B, D, E**). Note: red arrow of **C** granulate carinae of elytra; red arrow of **E** impressions and median sulcus of metaventrite.

**Figure 7.**
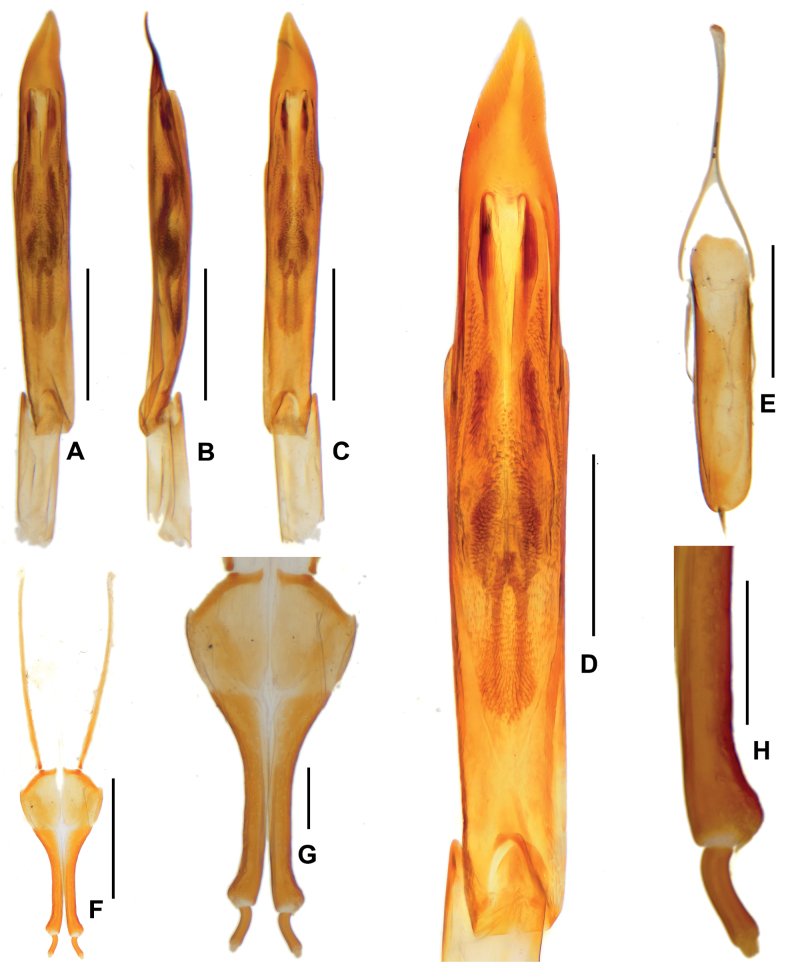
Diagnostic features of *Zaitzeviayingzuijieensis* sp. nov. **A** aedeagus, dorsal view **B** ditto, lateral view **C** ditto, ventral view **D** ditto, meidan lobe, dorsal view **E** sternite IX **F** ovipositor **G, H** apical part of ovipositor. Scale bars: 0.5 mm (**A–C, E, F**); 0.2 mm (**D, G**); 0.1 mm (**H**).

Figs 1C , 6 , 7 鹰嘴界寥溪泥甲

#### Type material

**(7** ♂, **1** ♀) **. *Holotype***: China: ♂, labeled ‘China: Hunan, Huaihua City (怀化市), Huitong County (会同县), Yingzuijie N. R. (鹰嘴界国家级自然保护区), light trap, H: ~400m, 02.08.2022, J.-H. Huang leg.’ (GUGC). ***Paratype***: 6 ♂♂, 1 ♀, with the same label data as the holotype (GUGC).

#### Description.

**Male. *Body elongately elliptical*** (Fig. 1C), dark brown or brown, legs brown, antennae and apical margin of labrum and pronotum reddish brown. Dorsal surface punctuated and shiny, covered with sparse setae. Plastron setae is confined to following areas: head (both dorsal and ventral surface, except clypeus), prosternum, outer part of elytra (include epipleura), outer parts of mesoventrite, metaventrite and abdomen (except median part) and surface of femora.

***Head*** (Fig. 6A), wider than long, surface covered with plastron setae and large, sparse punctures, each puncture bearing a longer seta, the interspaces between the punctures about twice the diameters of punctures. Clypeus evenly punctate with large punctures and covered with long, sparse setae, without plastron setae. Labrum transverse, about as wide as clypeus, covered with big punctures and long bristles at apical 2/3 portion, anterior margin finely rounded, anterolateral angles rounded. Antenna short, with eight antennomeres, antennomere I slightly longer than wide, with several short setae; antennomere II slightly longer than antennomere I, strongly expanded, covered with several long setae, apical margin circled with short setae; antennomere III longer than wide; antennomeres IV–VII strongly transverse; antennomere VIII elliptical, elongate and strongly expanded, apex covered with dense long setae.

***Pronotum*** (Fig. 6B) slightly wider than long, widest at base. Anterior margin arcuate with angles moderately produced and acute. Lateral margins finely curved. Basal margin trisinuate, emarginate before scutellum, posterior angles obtuse. Surface shiny, finely covered with large punctures, each puncture bearing a long seta; surface near apical angles microreticulate, setae on anterior half longer than on other parts. Longitudinal impression distinct but short, less than 1/3 length of pronotum, widest at basal 1/3; sublateral carinae short and shallow, from base to 1/3 of pronotum, near straight; a pair of small foveae located at middle of base of pronotum. Prosternal process (Fig. 6D) with rounded apex, disc shiny without punctures or setae, sides microreticulated.

***Elytra*** (Fig. 6C) about 1.70 times as long as wide, subparallel in basal 1/3, surface shiny and covered with rows of long, sparse setae. Each elytron with granulate carinae on strial intervals 5, 7, and 8; other intervals flat. Area from intervals to lateral margin covered with dense short setae. Hind wings well developed.

***Metaventrite*** (Fig. 6E), disc shiny, covered with large, sparse punctures, each bearing a long seta, without plastron setae, sides covered with plastron setae. Median sulcus long and distinct, extending from posterior margin to ca. 4/5 of metasternum, widest at base and get narrowed to apex, base of median sulcus with a pair of small round impressions. Areas along coxal cavities with a series of shallow and anomalous impressions.

***Disc of ventrites I–IV*** and anteriorly middle of ventrite V shiny, covered with sparse small punctures, without plastron setae, other areas of ventrites covered with plastron setae. Apical area of ventrite V granulated, apical margin distinctly emarginate at middle.

***Legs*** simple, femora swollen, surface covered with plastron setae; inner half of tibiae with cleaning fringes; tarsi about as long as tibiae; tarsal claws simple.

***Aedeagus*** (Fig. 7A–D), slender and elongate, median lobe nearly symmetrical, apex of median lobe cuspidal and distinctly asymmetrical, a pair of symmetrical and elongate sclerotizations located at apical 1/4 of median lobe, a pair of shorter and symmetrical sclerotizations located at basal 1/3 of median lobe. Sternite IX (Fig. 7E) with apical margin finely rounded, with a tuft of long setae, paraproct with basal round and expanded.

***Measurements***: CL: 2.33–2.38 mm; HL: 0.28–0.30 mm, HW: 0.40–0.41 mm; PL: 0.56–0.58 mm, PW: 0.74–0.76 mm; EL: 1.77–1.80 mm, EW: 0.91–0.93 mm.

**Female**: externally similar to the male, apex of sternite V rounded. Ovipositor as in Fig. 7F–H, stylus curved at base, apex with three short, finger-like sensilla; apex of coxite weakly roundly broadened at outer margin, with a curving sensilla; valvifer about 1.5 times as long as coxite, fibula distinctly curved at basal 1/2, base weakly expanded. Measurements: CL: 2.48 mm; HL: 0.33 mm, HW: 0.45 mm; PL: 0.65 mm, PW: 0.79 mm; EL: 1.83 mm, EW:0.96 mm.

**Figure 8.**
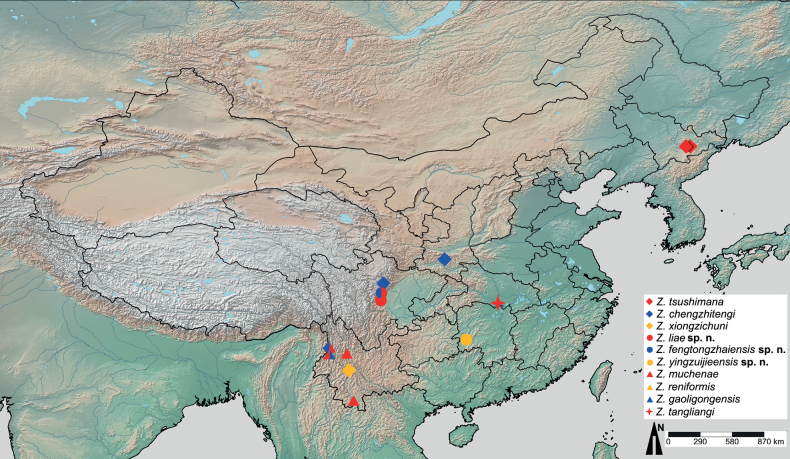
Distributional map of *Zaitzevia* species from the Chinese mainland.

#### Distribution.

China: Southwest Hunan Province.

**Figure 9.**
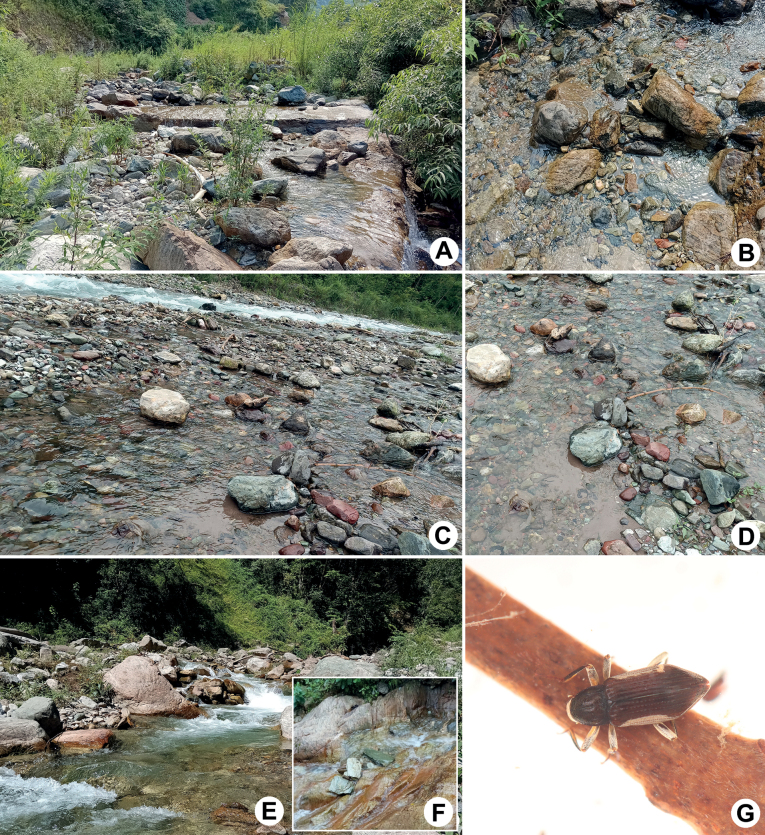
Habitat of *Zaitzevia* species **A** general environment of the type locality of *Z.sichuanensis* sp. nov.: Lengmugou Geopark **B** ditto, microenvironment **C** general environment of paratype: Xilingxueshan **D** ditto, microenvironment **E** general environment of the type locality of *Zaitzeviafengtongzhaiensis* sp. nov.: Fengtongzhai Nature Reserve **F** ditto, microenvironment **G** living adult of *Z.fengtongzhaiensis* sp. nov.

#### Biology.

All adults were collected by light trap. They might have similar habitat with other *Zaitzevia* species.

#### Etymology.

The specific epithet refers to the type locality, Yingzuijie Nature Reserve, Hunan Province; the name is treated as an adjective.

#### Comparative diagnosis.

The new species shares a similar habitus with several other species including the following: *Zaitzeviayaeyamana* Satô, 1963 from Japan, and *Z.tangliangi* Jiang & Wang, 2021. All species have a small body size (<3mm) and a very short, longitudinal impression on the pronotum. *Z.yingzuijieensis* sp. nov. can be distinguished from *Z.yaeyamana* by the following characters: 1) punctures on elytra much denser (vs sparser in *Z.yaeyamana*); 2) punctures on elytra much large (vs punctures on elytra very thin in *Z.yaeyamana*); 3) median lobe of aedeagus long and slender, about three times as long as phallobase (vs median lobe of aedeagus much stronger and shorter, about twice as long as phallobase in *Z.yaeyamana*). *Zaitzeviayingzuijieensis* sp. nov. can be distinguished from *Z.tangliangi* by the distinctly asymmetric apex of the median lobe of the aedeagus, while the apex of the median lobe is nearly symmetrical in *Z.tangliangi*. The sclerotizations near the apex of the median lobe is rounded at one end in the new species but pointed in *Z.tangliangi*.

### ﻿Key to *Zaitzevia* species from Chinese mainland

**Table:** 

1	Large species, CL > 3 mm	**2**
–	Smaller species, CL < 3 mm	**6**
2	Surface of elytra distinctly wrinkled; aedeagus short and strong, apex of median lobe of aedeagus characteristically arrowhead-like	***Z.chenzhitengi***
–	Surface of elytra shiny or only weakly wrinkled; aedeagus much slender	**3**
3	Apex of median lobe of aedeagus asymmetrically arrowhead-like, aedeagus curved at middle	***Z.sichuanensis* sp. nov.**
–	Apex of median lobe of aedeagus not arrowhead-like, middle of aedeagus not curved	**4**
4	Apex of median lobe of aedeagus widely triangular and strongly curved dorsally	***Z.muchenae***
–	Apex of median lobe of aedeagus cuspidal, not strongly curved	**5**
5	Surface of elytra weakly wrinkled; disc of prosternal process distinctly wrinkled; apical margin of sternite IX without tuft of setae	***Z.fengtongzhaiensis* sp. nov.**
–	Surface of elytra shiny; disc of prosternal process shiny, apical margin of sternite IX with tuft of long setae	***Z.xiongzichuni***
6	Longitudinal impression of pronotum extend from base of pronotum	***Z.tsushimana***
–	Longitudinal impression pronotum not contacted with base of pronotum	**7**
7	Longitudinal impression of pronotum long, 1/2 length of pronotum	***Z.gaoligongensis***
–	Longitudinal impression of pronotum short, about 1/3 length of pronotum	**8**
8	Sublateral carinae of pronotum long, 1/2 length of pronotum, apical half distinctly curved	***Z.reniformis***
–	Sublateral carinae of pronotum short, 1/3 length of pronotum, near straight	**9**
9	Disc of prosternal process wrinkled, apex of median lobe of aedeagus near symmetrical	***Z.tangliangi***
–	Disc of prosternal process shiny, apex of median lobe of aedeagus distinctly asymmetrical	***Z.yingzuijieensis* sp. nov.**

## Supplementary Material

XML Treatment for Zaitzevia sichuanensis

XML Treatment for Zaitzevia fengtongzhaiensis

XML Treatment for Zaitzevia yingzuijieensis

## ﻿Additional information

### Data availability

All of the data that support the findings of this study are available in the main text.
